# Functional and evolutionary analysis of alternatively spliced genes is consistent with an early eukaryotic origin of alternative splicing

**DOI:** 10.1186/1471-2148-7-188

**Published:** 2007-10-04

**Authors:** Manuel Irimia, Jakob Lewin Rukov, David Penny, Scott William Roy

**Affiliations:** 1 Allan Wilson Centre for Molecular Evolution and Ecology, Massey University, Palmerston North, New Zealand; 2 Departament de Genètica, Universitat de Barcelona, Barcelona, Spain; 3 Department of Molecular Biology, University of Copenhagen, Copenhagen, Denmark

## Abstract

**Background:**

Alternative splicing has been reported in various eukaryotic groups including plants, apicomplexans, diatoms, amoebae, animals and fungi. However, whether widespread alternative splicing has evolved independently in the different eukaryotic groups or was inherited from their last common ancestor, and may therefore predate multicellularity, is still unknown. To better understand the origin and evolution of alternative splicing and its usage in diverse organisms, we studied alternative splicing in 12 eukaryotic species, comparing rates of alternative splicing across genes of different functional classes, cellular locations, intron/exon structures and evolutionary origins.

**Results:**

For each species, we find that genes from most functional categories are alternatively spliced. Ancient genes (shared between animals, fungi and plants) show high levels of alternative splicing. Genes with products expressed in the nucleus or plasma membrane are generally more alternatively spliced while those expressed in extracellular location show less alternative splicing. We find a clear correspondence between incidence of alternative splicing and intron number per gene both within and between genomes. In general, we find several similarities in patterns of alternative splicing across these diverse eukaryotes.

**Conclusion:**

Along with previous studies indicating intron-rich genes with weak intron boundary consensus and complex spliceosomes in ancestral organisms, our results suggest that at least a simple form of alternative splicing may already have been present in the unicellular ancestor of plants, fungi and animals. A role for alternative splicing in the evolution of multicellularity then would largely have arisen by co-opting the preexisting process.

## Background

Alternative splicing (AS) of transcripts is common in diverse eukaryotic lineages. By this mechanism, a variety of transcripts and proteins are produced from a single gene, contributing to increased transcriptome and proteome diversity. AS has been reported in a wide range of eukaryotic groups including plants, apicomplexans, diatoms, amoebae, animals and fungi [1-5]. However, it is unclear and hard to assess whether this process has arisen independently in the different lineages (as suggested by some authors, e.g. [6]) or whether it was already present in their last common ancestor. The spliceosome, the machinery responsible for the splicing of introns in eukaryotic genes, is ancestral to all extant eukaryotic groups with the last common ancestor possessing a complex machinery, similar to that found in most modern organisms [7]. In addition, we recently argued that eukaryotic ancestors had weak 5' splice site boundary consensus sequences [8], a characteristic that is linked to the presence of AS in modern organisms [6]. These ancestral traits thus allow for the possibility that AS arose early in eukaryotic evolution.

How can we begin to address this issue? If AS arose independently in different lineages, we might expect that different classes of genes would show varying levels of AS in separate lineages, reflecting differential evolutionary histories. In particular, genes with regulatory functions, such as transcription factors [9,10] or signal transducers [11], exhibit high levels of alternative splicing in mammals, consistent with a central role for AS in generating the complexity of mammalian ontology [9], while basic enzymatic functions show less splice variation [9]. By the same reasoning, if AS arose along with the rise of organismal complexity in different multicellular lineages, genes central to this complexity would likely have high AS frequencies, while conserved ancient eukaryotic gene functions might have lower AS frequencies.

Notably, the finding of significant AS in the intron-rich pathogenic unicellular fungus *Cryptococcus neoformans *[3] demonstrates that widespread AS is not restricted to multicellular or highly-differentiated organisms. Indeed, from an evolutionary viewpoint, it is not likely that AS would evolve "in order for" multicellularity to develop; rather, it is possible that AS already existed (in at least a simple form) and was then later co-opted for multicellular development.

Intron/exon structure may be an important determinant for evolution of AS. Genes with more introns have more opportunities for AS. This would be consistent with vertebrates' higher intron numbers and AS frequencies [12]. However, recent results have shown that vertebrate intron number is not particularly high by historical metazoan standards [13-17], and that early eukaryotic ancestors likely harbored relatively high intron numbers [15,18-22].

We studied patterns of AS in 12 well-annotated genomes from plants, fungi and animals. We compared frequencies of AS of genes of different classes according to their gene structure, evolutionary origins, phylogenetic distribution and functionality. Our major findings include: (i) ancient genes (conserved in both plants and animals/fungi) are equally likely to have known AS as 'newer' genes; (ii) ancient functions are carried by genes that show relatively high levels of AS; (iii) genes found across all lineages (suggesting that they are essential for eukaryotic life) are no more likely to show AS than are genes that have been lost in one or more lineages; and (iv) there is a strong relationship between intron number and the existence of known AS across genes. We interpret our results to support the notion that a potentially widespread AS may have been present at least as early as the unicellular ancestor of animals, fungi and plants.

## Results

### Intron/exon structure and AS frequency

**Figure 1:**
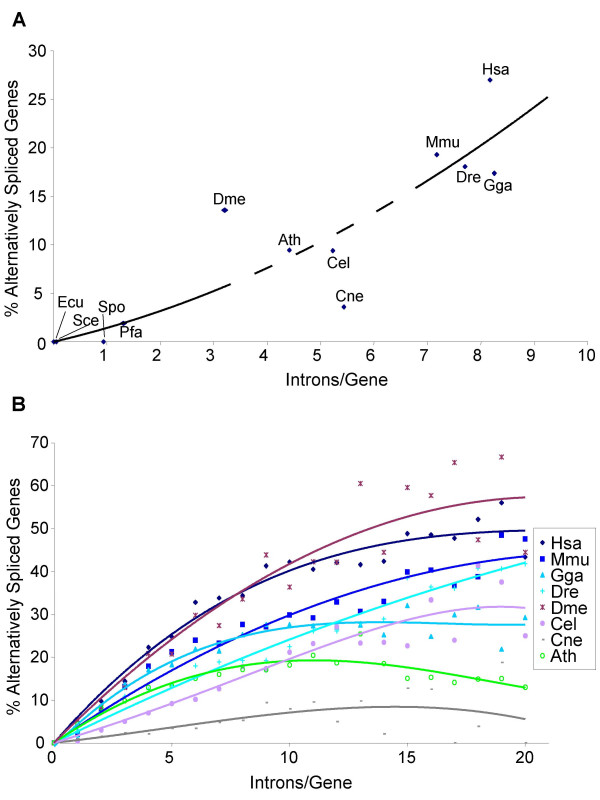
**Intron/exon numbers and AS frequency**. **A**: Percentage of alternatively spliced genes in different eukaryote genomes vs. the average number of introns per gene. Discontinuous line is an estimated interval for intron density of the ancestor of animals and plants (from 3.5 [18] to 7.0 [15]). **B**: Frequency of AS versus intron numbers per gene for the 8 species showing relatively high values of AS. Abbreviations: Hsa (*Homo sapiens*), Mmu (*Mus muscullus*), Gga (*Gallus gallus*), Dre (*Danio rerio*), Cel (*Caenorhabditis elegans*), Dme (*Drosophila melanogaster*), Ath (*Arabidopsis thaliana*), Sce (*Saccharomyces cerevisae*), Spo (*Schizosaccharomyces pombe*), Ecu (*Encephalitozoon cuniculi*), Pfa (*Plasmodium falciparum*), Cne (*Cryptococcus neoformans*).

We found a clear positive relationship between average intron number per gene and occurrence of AS across 12 animal, fungus, and plant species (Figure 1A). This relationship is also seen across genes within each of the eight species with significant frequencies of AS: higher intron number is associated with higher AS levels within each genome (Figure 1B). In particular, there is a steady increase in incidence of AS among genes with up to 6–10 introns. Given estimates of high intron densities in the plant animal ancestor (at least as high as modern *Caenorhabditis *species [15,18,23]), this finding is consistent with frequent ancestral AS.

### Age of alternatively spliced genes

**Figure 2:**
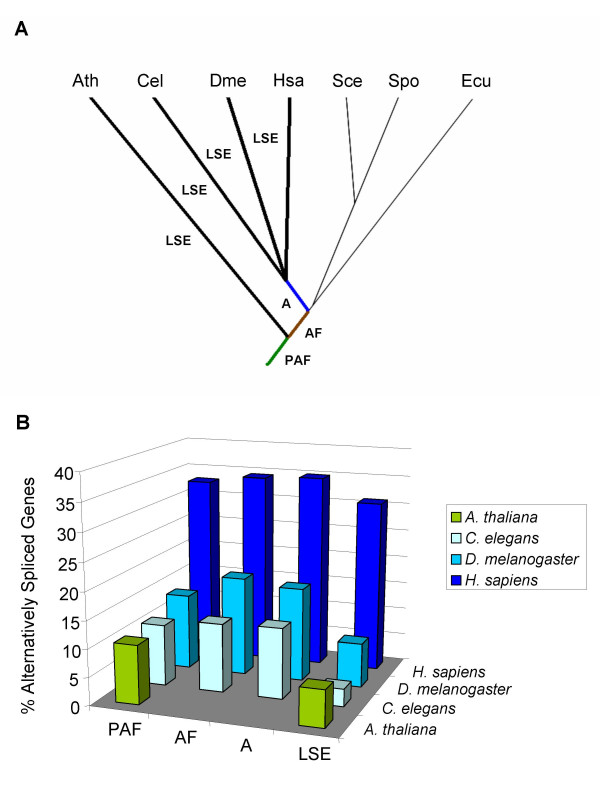
**Evolutionary origin of alternatively spliced genes**. **A**: Phylogenetic tree showing the relations between the seven species included in the KOG database and used in this study. PAF (green line) corresponds to the group of KOG's likely appeared before the split of animals, plants and fungi; AF (brown line), KOG's likely appeared in the fungamal ancestor; A (blue line), clusters of orthologous likely appeared in the ancestors of nematodes, insects and vertebrates; LSE's (four wide black lines) correspond to lineage specific expansions of plants, nematodes, insects and vertebrates. **B**: Percentage of AS for genes according to phylogenetic origin. PAF: ancestral to plants, animals and fungi. AF: ancestor of animals and fungi. A: animals. LSE: lineage specific expansions. Hsa (*Homo sapiens*), Cel (*Caenorhabditis elegans*), Dme (*Drosophila melanogaster*), Ath (*Arabidopsis thaliana*), Sce (*Saccharomyces cerevisae*), Spo (*Schizosaccharomyces pombe*), Ecu (*Encephalitozoon cuniculi*). Note that in *A. thaliana *genes can only group into PAF or LSE.

The KOG database [24] includes groups of orthologous genes for seven animal, fungus, and plant species. For each of the four species with significant AS levels in the KOG database (*H. sapiens, C. elegans, D. melanogaster *and *A. thaliana) *we divided gene families into four groups: 1) common (and thus presumably ancestral) to plants, animals, and fungi (PAF); 2) common to fungi and animals (AF); 3) specific to animals (A); 4) specific to a single lineage (LSE) (Figure 2A).

Figure 2B shows the percentage of genes in each group with known alternative splicing for each species. AS was found in all groups. LSE genes showed the lowest frequency of AS in each species (significantly lower than the whole set of genes in fly, worm and *Arabidopsis*, p < 0.0001 by Fisher exact tests), and AF and A genes showing the highest frequency. Interestingly, the most ancient group (PAF) showed relatively high levels of AS (significantly higher than the whole set of genes in worms and *Arabidopsis*, p < 0.0001 by Fisher exact test, and not significantly different to this set in humans and flies), indicating no constraints against evolution of AS in ancient eukaryotic genes. In particular, we identified 36 KOGs whose genes are highly alternatively spliced in all four species, which could reflect that these gene functions have been alternatively spliced in the plant/amimal ancestor (see Additional file 1).

### Gene dispensability and alternative splicing

**Figure 3:**
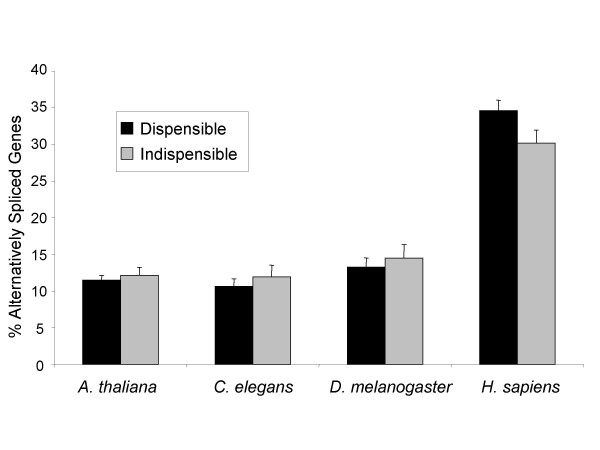
**Gene dispensability and alternative splicing**. Percentage of alternatively spliced genes according to gene dispensability in evolution. All the gene functions were present in the common ancestor of animals, plants and fungi. Dispensable genes (black): the KOG's to which they belong was lost in at least one of the animal or fungal species included in KOG database. Indispensable genes (grey): KOG's present in the seven studied species.

Among KOG's shared between *A. thaliana *and animals and/or fungi, we determined AS in 'indispensable' genes (those shared across all seven species in the KOG database) and 'dispensable' genes (absent from one or more opisthokonts); both classes of genes showed high AS levels (Figure 3). Furthermore, no correlation was found between a KOG's PGL (Propensity for Gene Loss, a measure of a gene's likelihood to be lost in evolution [25]) and AS in any species (data not shown).

Thus, genes encoding basic and highly conserved cellular functions are no less likely to be alternatively spliced than are other genes (for instance those involved in multicellularity or other complex functions).

### Cellular location of alternatively spliced genes

**Figure 4:**
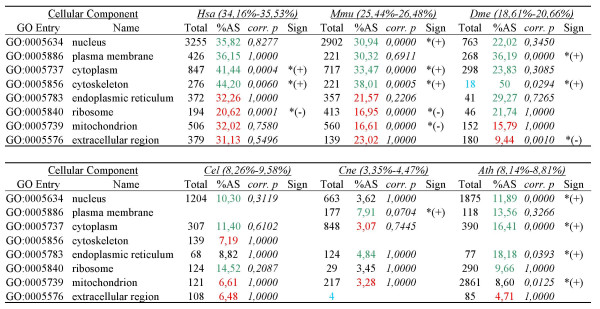
**AS frequency for GO categories for cellular locations**. For each category, green/red colored AS frequency indicates that the frequency is higher/lower than the average, with (*) denoting statistical significance. In the "Total" column, the total number of genes of each category is shown (categories represented by less than 35 genes are shown in blue). In parenthesis, for each species, 95% confidence interval for the average of alternatively spliced genes in all Cellular location categories. *p*-values are given after multiple testing correction. Abbreviations: Hsa (*H. sapiens*), Mmu (*M. musculus*), Dme (*D. melanogaster*), Cel (*C. elegans*), Cne (C. neoformans), Ath (*A. thaliana*).

The level of AS by cellular location for 6 eukaryotes is shown in Figure 4 and in detail in Additional file 2. Genes for proteins in most cellular locations showed AS. In particular, we were interested in the level of AS of genes encoding extracellular proteins, since many of these genes are likely to be important in the intercellular structures and communication vital to multicellularity (consistent with this notion, genes encoding extracellular products are much less frequent in the unicellular fungus *C. neoformans *(4/4578, 0.09%) than in the multicellular species (ranging from 0.32–3.19% across species)). Such extracellular-associated genes did not show elevated AS rates. Instead, genes encoding proteins located in the nucleus and plasma membrane generally have higher proportions of AS.

### Functional profile of alternatively spliced genes

**Figure 5:**
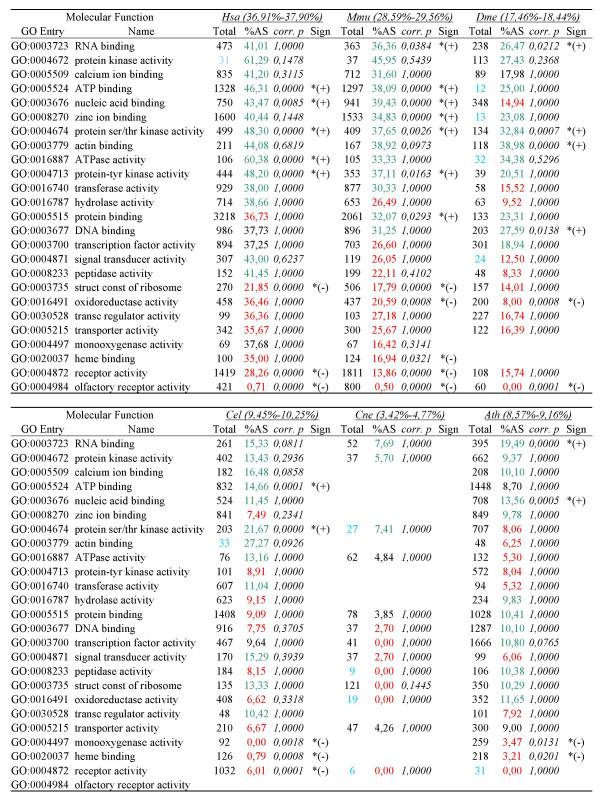
**AS frequency for GO categories for molecular function**. For each category, green/red colored AS frequency indicates that the frequency is higher/lower than the average, with (*) denoting statistical significance. In the "Total" column, the total number of genes of each category is shown (categories represented by less than 35 genes are shown in blue). In parenthesis, for each species, 95% confidence interval for the average of alternatively spliced genes in all Molecular Function categories. *p*-values are given after multiple testing correction. Abbreviations: Hsa (*H. sapiens*), Mmu (*M. musculus*), Dme (*D. melanogaster*), Cel (*C. elegans*), Cne (C. neoformans), Ath (*A. thaliana*).

**Figure 6:**
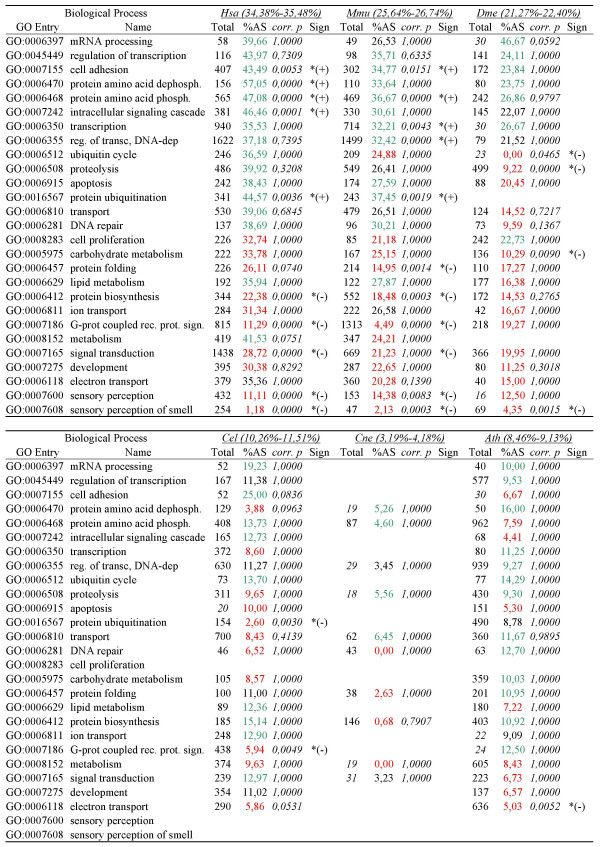
**AS frequency for GO categories for biological process**. For each category, green/red colored AS frequency indicates that the frequency is higher/lower than the average, with (*) denoting statistical significance. In the "Total" column, the total number of genes of each category is shown (categories represented by less than 35 genes are shown in blue). In parenthesis, for each species, 95% confidence interval for the average of alternatively spliced genes in all Biological process categories. *p*-values are given after multiple testing correction. Abbreviations: Hsa (*H. sapiens*), Mmu (*M. musculus*), Dme (*D. melanogaster*), Cel (*C. elegans*), Cne (C. neoformans), Ath (*A. thaliana*).

AS levels across species for molecular function (F) and biological process (P) GO categories are shown in Figures 5 and 6, respectively, and in detail in Additional files 3 and 4, respectively. Again, gene categories generally associated with multicellularity (development, sensory-related functions) did not show elevated AS rates. Among molecular functions, protein kinase activity, RNA binding and calcium ion binding generally had high AS frequencies while monooxygenase activity, receptor activity, transporter activity and heme binding had much lower AS across all species. Biological processes showed greater variation across species

Functional profiling of alternatively spliced genes thus shows that most genes encoding most cellular functions exhibit AS. Some functions seem to be especially amenable to AS, perhaps due to these gene functions being particularly improved by the production of multiple products. AS is particularly prevalent in genes associated with regulation and signaling, consistent with previous observations [9,11]. Interestingly, the overrepresentation of AS in genes with regulatory functions previously observed in mammals is observed across lineages.

## Discussion

### Patterns of genome-wide AS usage are similar in different eukaryotic lineages

We studied patterns of genome-wide AS in 12 eukaryotic genomes. Our major findings include:

1) a correspondence between intron number and frequency of known AS, both within and between species;

2) evidence that ancient genes show relatively high levels of known AS.

3) no evidence for elevated AS in recently evolved genes – in fact, the most recently evolved genes are less likely to have known AS;

4) no clear evidence for elevated AS in classes of genes thought to be important in the rise of multicellularity;

5) a variety of similarities in the patterns of AS across diverse species, which could reflect patterns inherited from a putative AS-rich plant-animal ancestor.

Alternatively spliced genes in animals, plants and fungi have several common features. In all studied species, AS is widely found across genes with different functions, although those associated with regulation (i.e. protein kinase activity or RNA binding) show consistently higher AS levels. Also, the species studied showed similar patterns of AS usage in genes of different evolutionary age. Finally, intron number per gene was related with AS frequency in a similar manner in all species.

The simplest hypothesis to explain these similarities is that AS is homologous in these groups, inherited from their common ancestor, and that AS patterns in the common ancestor might have been similar. However, caution is necessary in interpreting this result. Given the high rates of gains and losses of AS events, the alternative hypothesis of convergent evolution of AS patterns in the different lineages, although less parsimonious, cannot be excluded.

### Ancient genes and functions show relatively high levels of AS

Also consistent with a relatively early origin of AS is our finding that recently evolved gene families (LSE) showed the lowest frequency of AS in all species (Figure 2B). Many of these genes are likely associated with newly evolved and complex lineage-specific traits in plants and animals. On the other hand, ancient gene families (PAF), which were already present in the ancestor, are highly alternatively spliced in modern organisms (Figure 2B) and a wide range of fundamental ancient functions are currently performed in eukaryotic cells by alternatively spliced genes (Figures 5 and 6), indicating no constraints for ancient genes to be alternatively spliced. Finally, gene functions that show consistently higher (e.g. RNA binding and protein kinase activity) or lower (e.g. mono-oxygenase activity) levels of AS across eukaryotes could have also had similar relative levels in the common ancestor.

### Alternatively splicing and proteomic networks

We found no clear relationship between incidence of AS and gene dispensability (Figure 3). Indispensable genes tend to have large numbers of interaction partners, occupying central positions in proteomic networks, while dispensable genes usually occupy external positions in the interacting networks [25]. These results thus suggest that AS may be integrated across all levels of eukaryotic proteomic networks.

### Intron-rich gene structures is the main requirement for AS

We show that intron/exon number correlates strongly with the frequency of AS within and between genomes (Figure 1). In accordance, most intron-reduced genomes, such as those of most microsporidia and ascomycetes, show no AS [6] and other relatively reduced genomes, such as amoebas [26] or apicomplexans [27] do not exhibit high frequencies of AS. Interestingly, LSE genes, found to have significantly lower levels of AS, show lower average intron numbers than the other groups.

Our and others' previous work has shown that the plant-animal ancestor was at least moderately intron-rich (with at least as many introns as modern *Caenorhabditis *species), and that lower modern densities in some lineages reflect widespread intron loss [15,19,21,23,28-30]. Taken with present results, there are two important potential implications. First, retention of ancestral intron densities was likely an important condition for modern AS. Thus, if in fact AS played an important role in the emergence of organismal complexity [31,32], differential retention of ancestral introns would have profound consequences for morphological evolution across lineages.

Second, intron-rich ancestors are likely to have had significant AS. All thoroughly studied intron-rich genomes show relatively high frequencies of AS, suggesting both that a complex gene structure favors AS and that AS could have an important role in most non-reduced genomes, with high numbers of introns per gene. As we mention above, this is especially interesting in light of accumulating evidence that the last common ancestor of plants and opisthokonts was at least moderately intron rich [15,18-22] (with an estimated intron density between ~3.5 [18] and ~7.0 [15] introns per gene) and that it had weak consensus 5' splice site boundaries [8]. Among modern eukaryotes, both high intron number and weak 5'ss are characteristic of diverse species with widespread alternative splicing [8]. Therefore, these studies together strongly suggest the presence of AS in plant-animal ancestor

### Intron numbers, spliceosomal errors, functionality and origin of AS

It is important to note that our present results do not address the functionality of alternative splice variants (and thus of AS) either in early eukaryotes or in modern organisms, a topic currently under debate [33,34]. Alternative transcripts produced from the same gene might: (i) encode different functions, (ii) reflect nonfunctional (but common) variants or (iii) represent rare spliceosomal errors, which will all appear in EST databases and thus in EST-based AS annotations. It seems likely that all three cases contribute to modern transcriptome variability. If in fact our argument is correct (that early eukaryotes already utilized extensive AS), it would be interesting to know how levels of AS functionality have changed through time. Increased requirements on proteome and regulatory flexibility could have driven an increase in functional AS. In tandem, refinements in the spliceosomal machinery could have increased splicing fidelity through eukaryotic evolution, disproportionately decreasing nonfunctional AS variants.

Interestingly, the evolutionary origin of functional AS is likely related to mis-splicing (splicing errors). AS might have evolved from mis-splicing as the early eukaryotic cells evolved to use and benefit from multiple splicing outputs. Therefore, the widespread production of multiple splice forms could be a main requirement for the origin of functional AS. Thus, our results along with the likely existence of weak splice sites in early eukaryotes [8] do not prove that early eukaryotes had functional AS, but they strongly suggest that the last plant-animal ancestor had at least such additional splice variants available for potential participation and recruitment in biological processes.

### AS in unicellular organisms and the origin of multicellularity

A striking potential implication of our results is that AS already existed in the plant-animal ancestor, a rather ancient and "primitive" unicellular eukaryote. As seen in some modern unicellular organisms (e.g. *Cryptococcus*), AS could have played an extensive role in the biology and evolution of these ancestral unicellular eukaryotes.

In this case, AS would have predated multicellularity and could perhaps have been recruited to allow the rise of multicellular complexity. This would resemble other biological processes, like apoptosis, whose origin precedes the rise of multicellular organisms, although the co-option of this ability was crucial for the advent of multicellularity [35].

### Reliability of AS databases to answer evolutionary questions

Though we restricted our analysis to well-annotated genomes from long- and deeply-studied species with wide cDNA/EST coverage [36], it is likely that many alternative transcripts are not represented in current annotations, introducing the possibility of sampling biases. For instance, some gene types have been more thoroughly studied, and therefore may show higher proportions of annotated AS. However, such differences are unlikely to explain our central conclusions, since they are largely based on shared similarities, not differences (similar incidence of alternative splicing in old and new genes, dispensable and indispensable genes, genes of different functional classes), and clear associations across large numbers of genes. Indeed, EST coverage in *C. elegans *does not correlate with fractions of predicted alternatively spliced genes across different GO categories or gene ages, suggesting that the observed patterns are not due to EST sampling (Additional file 5).

Another potential problem associated with EST based annotation is the source of these data, especially in the case of humans. Many human EST libraries derive from cancerous or abnormal cell lineages, thought to contain aberrant, disease related alternative splice variants [37]. If these variants are more frequent among some groups of genes, this could introduce a bias in our results. However, our results for functional ontologies are based on data from a variety of species and the patterns presented here are consistent among all the species despite this potential source of noise in human databases.

We used genome annotation databases for this analysis because they are constructed using very similar approaches and so they might be more suitable for comparing these species. Supporting the quality of the studies databases, we found in the thorough analysis of the *C. elegans *Wormbase dataset that the vast majority of alternatively spliced isoforms included are well supported by experimental evidence, and only very few cases represent annotation mistakes.

Finally, it should be noted that the current results concern only presence/absence of AS, rather than number of alternative transcripts. Since alternatively spliced genes may produce from 2 to hundreds of isoforms, the effects on the transcriptome output will be quite different across genes, and further studies should address this important issue. Instead, we have concentrated on known AS presence/absence in a gene, which is likely to be less sensitive to differences in EST sampling. Similarly, different positions of the AS events in each gene may produce very different outputs with different effects on the organism's fitness. Our analysis does not take differences in function between splice variants into account. However, these considerations are unlikely to affect our conclusions about AS in early eukaryotes.

To verify our hypotheses on the emergence of AS, further studies of conservation of AS mechanisms (i.e. use of splicing regulators), splicing boundaries, and expression patterns will be necessary. Characterization of levels and patterns of AS in diverse additional eukaryotes, particularly unicellular intron-rich species, will also be important. Species of apicomplexans [4] and diatoms [5] have already been shown to have AS. EST and genome sequencing projects will provide data to assess whether AS was an ancestral feature of eukaryotic organisms, playing another important role in the complex RNA processing of early eukaryotes [7,38].

## Conclusion

We find similar patterns of genome-wide AS usage in different eukaryotic lineages. We show that ancient genes and functions (present in the common ancestor of plants and opisthokonts) have high levels of AS in modern organisms indicating no bias against AS of these genes. These genes were also likely intron-rich in the common ancestor [15,18-22], which we find to be the main requirement for AS. Since the spliceosomal machinery is widely conserved throughout eukaryotes [7,38], our results favor the hypothesis that some form of AS appeared relatively early in eukaryotic evolution, at least in the unicellular common ancestor of plants, animals and fungi (around 1300 million years ago [39,40], quite early in the evolution of extant eukaryotes [41]). This implies AS appeared before the rise of multicellular organisms, and could therefore have an important role in the biology of ancient unicellular organisms.

## Methods

### Datasets and resources

GenBank genome annotations were downloaded from NCBI webpage [42] or Ensembl database [43] for six metazoa: human (*Homo sapiens *(NCBI 36 Ensembl 38.36)), mouse (*Mus musculus *(NCBIm35 Ensembl 38.35)), chicken (*Gallus gallus *(WASHUC1 Ensembl 38.1n)), zebra fish (*Danio rerio *(Zv5 Ensembl 38.35e)), fruitfly (*Drosophila melanogaster *(FlyBase release 4.1)), worm (*Caenorhabditis elegans *(WS150 Wormbase 38.150a)); four fungi: *Cryptococcus neoformans *B3501-A (NC_006670, NC_006679–NC_006687, NC006691–NC006694), *Schizosaccharomyces pombe *972h (AL672256-8.1), *Saccharomyces cerevisiae *YJM789 (AAFW00000000.1), and *Encephalitozoon cuniculi *GB-M1 (AL391737.1, AL590442-50.1); one plant: *Arabidopsis thaliana *(NC_003070.5, NC_003071.3, NC_003074.4, NC_003075.3, NC_003076.4, based on TAIR genome annotations); and one apicomplexan: *Plasmodium falciparum *HB3 (AANS00000000.1).

### Quality of the databases

For many species, there are currently various genomic databases available having information on AS. In each such case, we used the richest and most up-to-date database, containing the largest number of described alternatively spliced isoforms. Each of the databases was constructed by automatic predictions of gene structures, generally combining different software, and then confirmed by mapping ESTs and cDNAs onto genomic sequences and usually manually curated. Described alternatively spliced isoforms are based on alignments of ESTs and cDNAs onto these gene models. For each genome, some subsets of genes are manually annotated and thoroughly studied. Detailed explanations of the methods using in deriving these databases are available from the primary references [3,44-46] and from the Ensembl, TAIR and NCBI web pages.

To better understand these genome annotations we further explored one of them, the Wormbase annotation of *C. elegans*. We studied each gene that was annotated to be alternatively spliced. We found that 97.8% of the isoforms had one or more kinds of experimental support (RNA, ORF sequence tags (OSTs) and/or ESTs), described as "confirmed by cDNA(s)" or "partially confirmed by cDNA(s)", thus only ~2.2% of isoforms are predictions. In addition, for each case we aligned the different isoforms against the genomic sequence. In only 2.4% of cases, we found that slight errors, generally one or two base indels, were responsible for the annotation of alternative splicing.

To test the effects of sampling biases we analyzed the coverage of ESTs per Kb for each gene and for each category of gene. For each gene we counted the number of matching ESTs per gene available in Wormbase and divided it by the length of the longest transcript. Importantly, no correlation was found between EST coverage and percentage of genes that were alternatively spliced for any of the GO classifications (cellular location, molecular function, or biological process), or for age of gene (Additional file 4).

### Evolutionary analyses

For evolutionary analyses we used the Eukaryotic Clusters of Orthologous Groups (KOGs), which includes putative ortholog sets for seven species: *Homo sapiens, Caenorhabditis elegans, Drosophila melanogaster, Arabidopsis thaliana*, *Saccharomyces cerevisae*, *Schizosaccharomyces pombe *and *Encephalitozoon cuniculi*. This database is suitable for the study of protein functions from an evolutionary perspective, addressing issues such as origin of gene functions or their dispensability during eukaryotic evolution [47]. Data were downloaded from the corresponding NCBI webpage [48] and linked to current genome annotation databases. Data was carefully filtered for repetitions resulting from database linking and from the updating of annotations of the genes included in the KOG database.

### Gene Ontology analyses

Gene Ontology annotations for cellular location (C), molecular function (F) and biological process (P) for genes from *H. sapiens*, *M. musculus, C. elegans, D. melanogaster, C. neoformans *and *A. thaliana *were obtained from Gene Ontology Consortium website [49]. This database was linked to Ensembl or NCBI gene ID's, using UniProt ID's [50], if necessary. Data was carefully filtered to avoid redundancies due to database linking. We analyzed in *H. sapiens *a total of 18589 entries in 458 C-GO categories, 36653 entries in 2063 F-GO categories and 29162 in 1867 P-GO categories; in *M. musculus*, 19002 entries in 450 C-GO categories, 33461 entries in 1898 F-GO categories and 24793 in 2119 P-GO categories; in *D. melanogaster*, 5811 entries in 411 C-GO categories, 11916 in 1571 F-GO categories and 20307 in 1657 P-GO categories; in *C. elegans*, 7151 entries in 174 C-GO categories, 21627 in 837 F-GO categories and 9579 in 562 P-GO categories; in *C. neoformans *4578 entries in 279 C-GO categories, 3298 in 969 F-GO categories and 5564 in 884 P-GO categories; in *A. thaliana*, 26592 entries in 281 C-GO categories, 34959 in 1255 F-GO categories and 27845 in 1210 P-GO categories.

### Statistical analysis

Percentages of alternatively spliced genes were calculated for each category under study. The correspondent 95% confidence interval (CI) was calculated for each percentage using the standard formula: a±1.96a(1−a)N
 MathType@MTEF@5@5@+=feaafiart1ev1aaatCvAUfKttLearuWrP9MDH5MBPbIqV92AaeXatLxBI9gBaebbnrfifHhDYfgasaacH8akY=wiFfYdH8Gipec8Eeeu0xXdbba9frFj0=OqFfea0dXdd9vqai=hGuQ8kuc9pgc9s8qqaq=dirpe0xb9q8qiLsFr0=vr0=vr0dc8meaabaqaciaacaGaaeqabaqabeGadaaakeaacqWGHbqycqGHXcqScqaIXaqmcqGGUaGlcqaI5aqocqaI2aGndaGcaaqaamaalaaabaGaemyyaeMaeiikaGIaeGymaeJaeyOeI0IaemyyaeMaeiykaKcabaGaemOta4eaaaWcbeaaaaa@3B28@, where *a *is the fraction of alternatively spliced genes in a given group and *N *the total number of genes in that group.

To assess statistical under/overrepresentation of AS in each studied category, we used Fisher exact tests (assuming the one-sided probability for similarity of samples). For each of the three blocks of GO terms presented in Figures 4, 5 and 6 (cellular location, molecular function and biologcial process, respectively), we corrected for multiple testing using full Bonferroni correction.

In the Additional files 2, 3 and 4, we excluded groups of genes that contained less than 35 genes.

### Analysis of alternative isoforms

Gene, intron and exon information was extracted from their annotation using a PERL script "Intron_finder.pl" as previously described [36]. For each gene, custom PERL scripts assessed intron number and alternative splicing (i.e. the position or length of any alignable intron or internal exon is different in at least two different isoforms). AS events in *P. falciparum *were extracted from [51].

## Competing interests

The author(s) declares that there are no competing interests.

## Authors' contributions

MI carried out the data collection, genomic and statistical analyses, designed and conceived the study and drafted the manuscript. SWR and JLR helped to draft the manuscript and participated in the interpretation and analyses of the data. DP participated in the design of the study, coordination and helped to draft the manuscript. All authors read and approved the final manuscript.

## Supplementary Material

Additional file 1 **Ancient alternatively spliced KOG's**. List of 36 ancient KOG (appeared before the split of animals, fungi and plants) that show high AS incidence in *A. thaliana*, *D. melanogaster*, *C. elegans *and *H. sapiens*. Click here for file

Additional file 2 **Cellular locations and alternative splicing**. List of different cellular locations and their AS frequency for *A. thaliana*, *D. melanogaster*, *C. elegans, C. neoformans, M. musculus *and *H. sapiens*. Click here for file

Additional file 3 **Molecular functions and alternative splicing**. List of different molecular functions and their AS frequency for *A. thaliana*, *D. melanogaster*, *C. elegans, C. neoformans, M. musculus *and *H. sapiens*. Click here for file

Additional file 4 **Biological Processes and alternative splicing**. List of different biological processes and their AS frequency for *A. thaliana*, *D. melanogaster*, *C. elegans, C. neoformans, M. musculus *and *H. sapiens*. Click here for file

Additional file 5 **EST/cDNAs Sampling Bias Control**. Control for EST/cDNAs sampling bias. It has been performed in *C. elegans*. The document contains 4 figures, corresponding to: cellular locations (C), molecular functions (F), biological process (P) and species groups. Click here for file
