# Supplementary material for: Functional and evolutionary analysis of alternatively spliced genes is consistent with an early eukaryotic origin of alternative splicing
Source: BMC Evol Biol. 2007 Oct 4;7:188. doi: 10.1186/1471-2148-7-188 (PMC2082043; doi:10.1186/1471-2148-7-188)

For each Figure:

- Blue: Percentage of AS per category.
- Pink: average number of ESTs per Kb per category.

Cellular location (C)

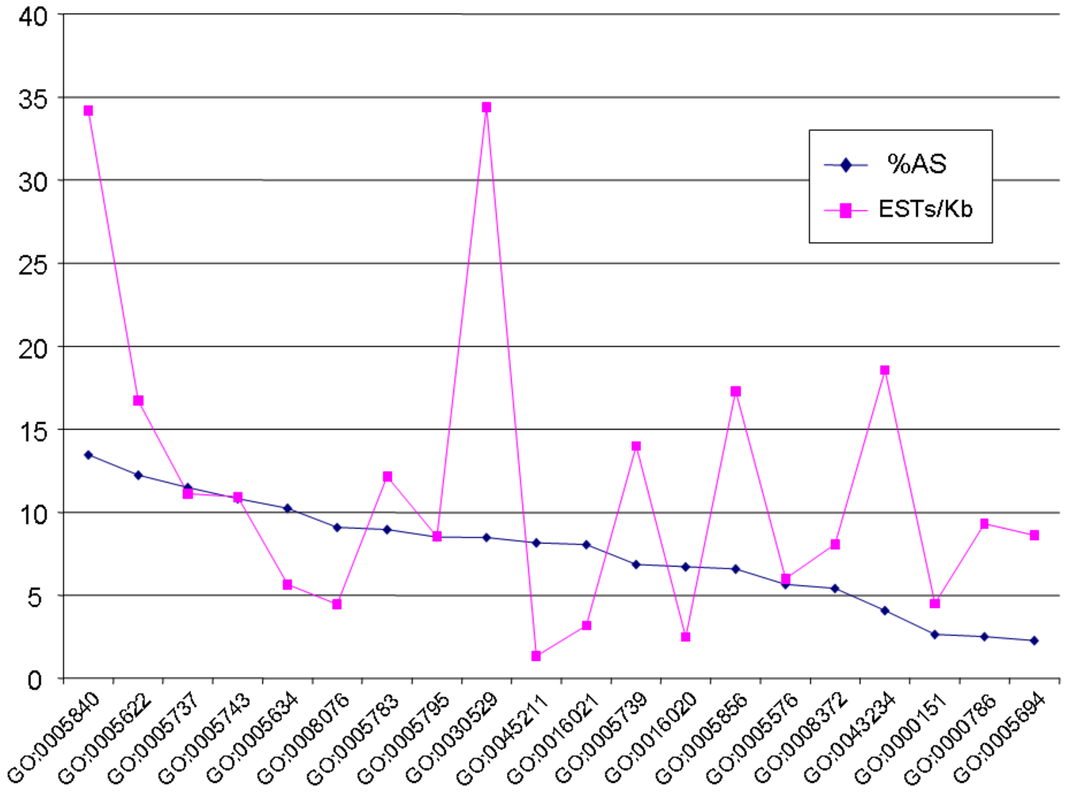

Molecular Functions (F)

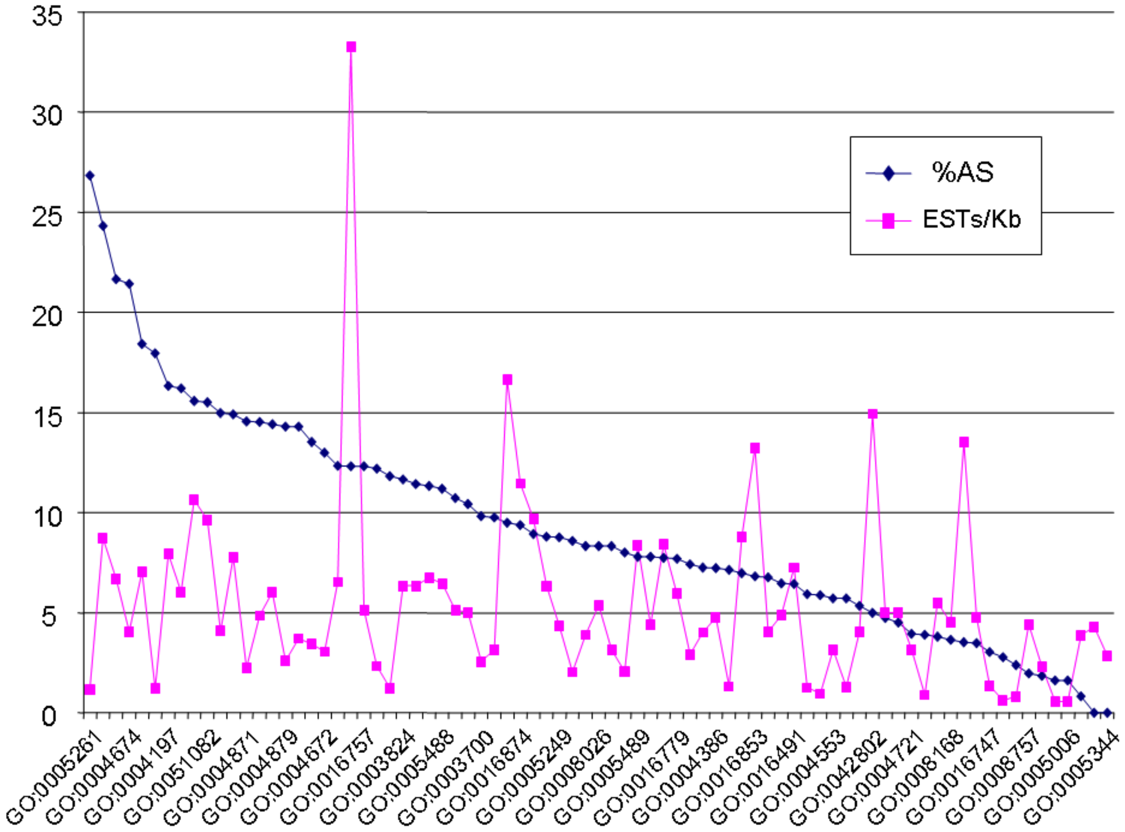

Biological Process (P)

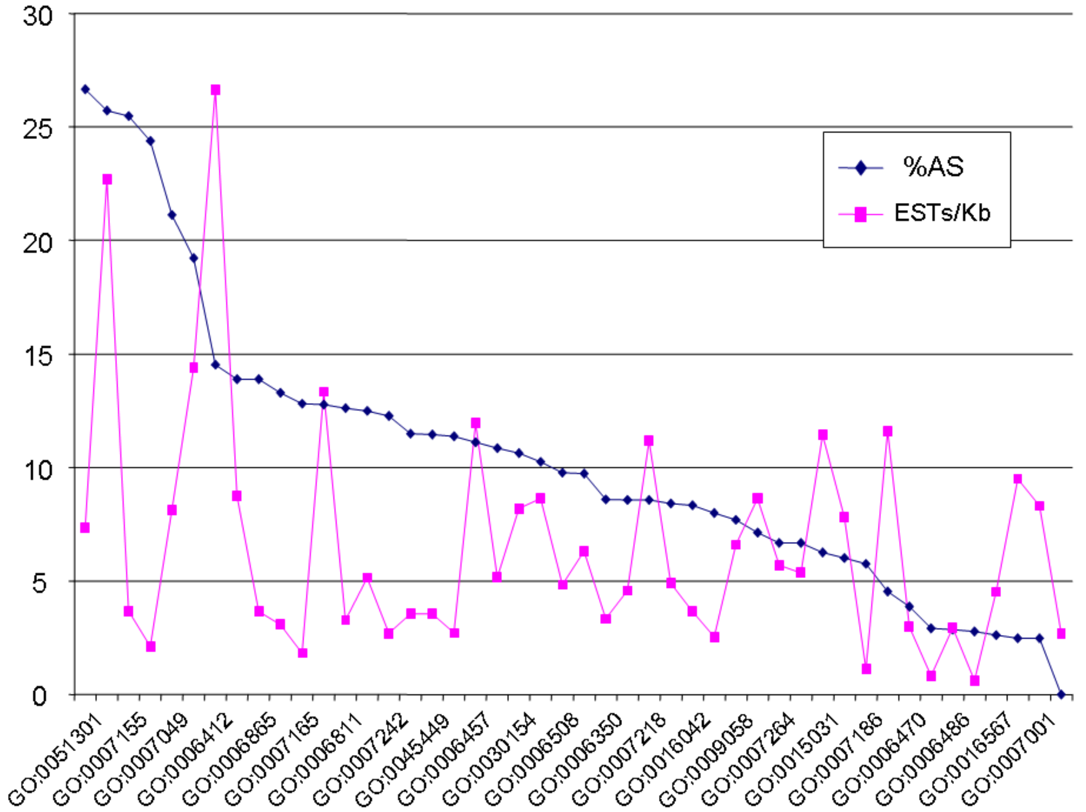

Species groups

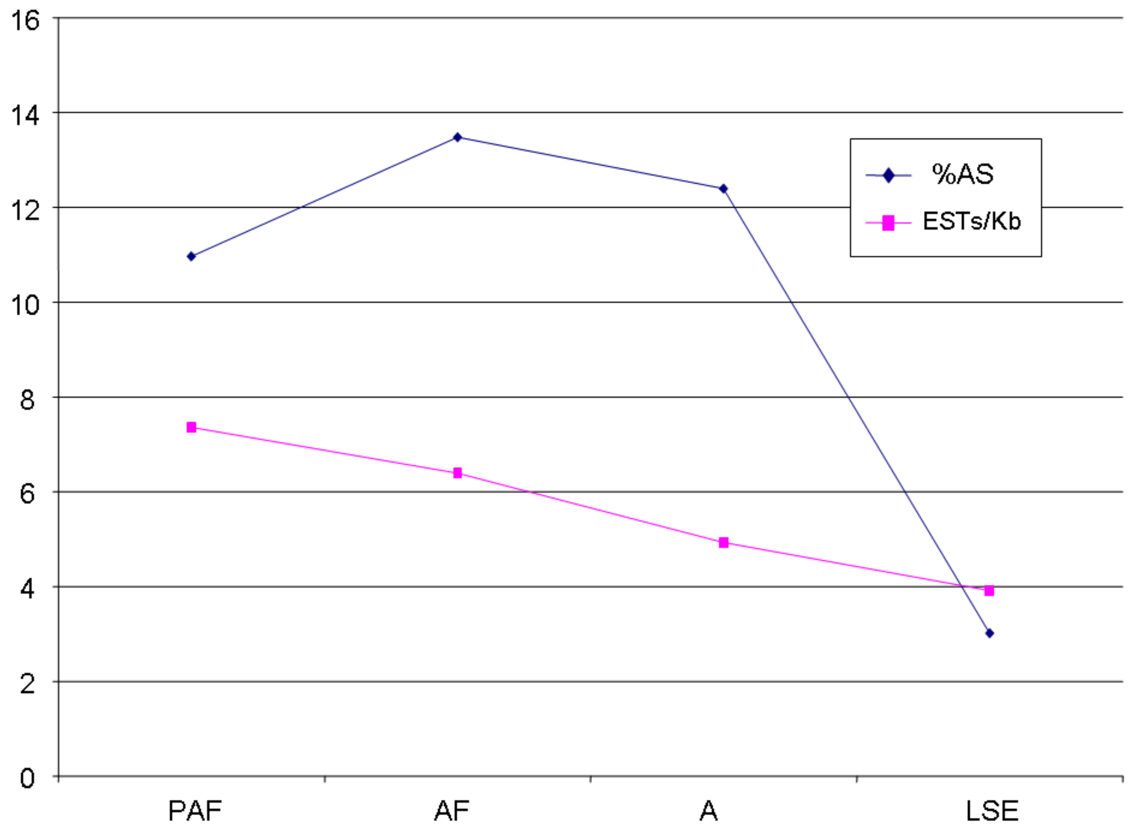

Supplement: Additional file 5 — EST/cDNAs Sampling Bias Control. Control for EST/cDNAs sampling bias. It has been performed in C. elegans. The document contains 4 figures, corresponding to: cellular locations (C), molecular functions (F), biological process (P) and species groups. [file 1471-2148-7-188-S5.pdf]
